# Phenotypic Variability in Camurati–Engelmann Disease: A Case Report of a Family with the c.653G>A Pathogenic Variant in the *TGFB1* Gene

**DOI:** 10.3390/genes15111354

**Published:** 2024-10-22

**Authors:** Talyta Campos, Elza Uchoa, Victor Santos, Raffael Zatarin, Rosenelle Benício, Clayson Gomes, Aparecido da Cruz

**Affiliations:** 1 Replicon Research Nucleus, Graduate Program in Genetics, School of Medical and Life Sciences, Pontifical Catholic University of Goiás, Goiânia 74605-050, GO, Brazil; campostalytabmd@gmail.com (T.C.);; 2 Clinical Genetics Service, Center for Rehabilitation and Readaptation Dr. Henrique Santillo, State Health Secretary of Goiás, Goiânia 74605-050, GO, Brazil; 3 Graduate Program in Genetics and Molecular Biology, Federal University of Goiás, Goiânia 74605-050, GO, Brazil

**Keywords:** hyperostosis, autosomal dominant, bone metabolism, rare disease

## Abstract

Camurati–Engelmann Disease (CED), or Progressive Diaphyseal Dysplasia, is a rare autosomal dominant disorder caused by heterozygous mutations in the *TGFB1* Gene, essential for bone regeneration. This study examines the genotype–phenotype relationship in a family diagnosed with CED, specifically focusing on a missense variant (c.653G>A, p.Arg218Cys). The family comprised a mother and her two children, all of whom were found to carry the same disease-causing variant. The second child exhibited severe symptoms by age six, including progressive weakness and joint pain, leading to wheelchair dependency. The mother displayed milder symptoms with preserved independence. The firstborn son, initially asymptomatic, developed gait abnormalities and pain during adolescence. Clinical evaluations revealed characteristic hyperostosis of long bones, with significant variability in symptom onset and severity among family members, potentially indicative of genetic anticipation. This case underscores the importance of genetic testing and interdisciplinary management in CED, as traditional treatments, including corticosteroids and NSAIDs, often yield limited efficacy and notable side effects. Our findings contribute to the understanding of CED’s pathophysiology and highlight the necessity for tailored therapeutic approaches. The identification of the common *TGFB1* variant in this family reinforces the critical role of *TGFB1* in bone metabolism and suggests avenues for further research into targeted therapies. Such reports enhance awareness and provide valuable insights for healthcare professionals managing rare genetic disorders.

## 1. Introduction

Camurati–Engelmann Disease (CED), also known as Progressive Diaphyseal Dysplasia (OMIM #131300), is a rare condition inherited in an autosomal dominant (AD) pattern, caused by heterozygous variants in the Transforming Growth Factor Beta 1 (TGFB1) gene, which plays a crucial role in bone regeneration [1,2]. Mário Camurati began studying the disease in 1922, and Walter Engelmann reported the first case in 1929. To date, it is estimated that over 300 cases have been reported worldwide [3]. While the prevalence of CED is not well known, Cui et al. [4] estimated an occurrence of 1 in 1,000,000 individuals, although its penetrance remains unknown [5].

CED is characterized by hyperostosis of the diaphysis of long bones and the skull, affecting both the periosteal and endosteal surfaces. Involvement of the pelvic bones, ribs, spine, and bones of the feet and hands has also been described. The primary symptoms leading to clinical hypothesis of CED include proximal muscle weakness, fatigue, bone pain (especially in the lower limbs), a waddling gait, hearing loss, and headaches [5,6]. Other phenotypic manifestations that may occur include visual impairment, exophthalmos, visual field defects, facial paralysis, anorexia, slender limbs, joint contractures, hepatosplenomegaly, decreased subcutaneous fat, delayed puberty, hypogonadism, macrocephaly, prominent forehead, ptosis, cranial nerve paralysis, hydrocephalus, genu valgum, and anemia [5,7,8]. The onset of symptoms varies and can occur from birth to late adulthood [9].

The *TGFB1* Gene, located on chromosome 19q13.2, contains seven exons, and encodes the TGFB1 protein, which consists of 390 amino acids. In its mature form, this protein plays a role in regulating cell proliferation, differentiation, migration, and apoptosis [5]. Activated TGFB1 is crucial for bone deposition, regulating the functions of osteoblasts and osteoclasts, and maintaining the balance of bone metabolism in the body [10]. Pathogenic variants in this gene can lead to increased bone formation and resorption, as they destabilize the peptide complex and hyperactivate the Transforming Growth Factor (TGF) signaling pathway, potentially leading to increased intramembranous bone formation. The increase in bone mass results in symmetrical skeletal thickening, primarily affecting long bones such as the femur and tibia [7].

The nosological diagnosis can be made through clinical investigation of symptoms, characteristic radiological findings, and molecular genetic testing. Following the diagnosis, symptom management is tailored to the needs of each individual, typically involving medications such as corticosteroids, bisphosphonates, Non-Steroidal Anti-Inflammatory Drugs (NSAIDs), and losartan to alleviate bone pain and improve fatigue and muscle weakness. Evaluations by specialized professionals are recommended to investigate all possible phenotypes of the condition and to prevent the emergence of phenotypes that have not yet manifested [6,11].

This report aims to describe the genotype–phenotype relationship in three patients from the same family diagnosed with Camurati–Engelmann Disease. Such case reports are significant as they can highlight unusual patterns, varied clinical presentations, or a novel disease-causing variant associated with the disease, thereby aiding researchers and healthcare professionals in understanding its pathophysiology and developing new approaches for diagnosis, treatment, management, and monitoring. Additionally, for patients and families affected by rare diseases, reading similar case reports can provide a sense of community and validation, helping them feel less isolated by showing that others face similar challenges. The research in question has been submitted and approved by the ethics committee of the Pontifical Catholic University of Goiás (PUC-GO) under approval number 6.057.949.

## 2. Case Description

A family consisting of a mother and two children was evaluated by the Medical Genetics and Multidisciplinary Department at the Dr. Henrique Santillo State Rehabilitation and Readaptation Center (CRER) in Goiânia, Goiás, Brazil. The family, originally from Goiânia, was residing in Goianira-Goiás at the time of the clinical evaluation. Initially, only the second-born child exhibited symptoms, prompting genetic testing that identified a variant in the *TGFB1* Gene, leading to the diagnosis of Camurati–Engelmann Disease. Subsequently, the same variant was found in both the mother and the proband’s sibling.

### 2.1. Patient No. 1 (Proband/Second-Born Child)

The mother, G2P2A0, reported no history of miscarriages or consanguinity. The pregnancy with the second child, a male, was uneventful, and was delivered by caesarean section at term, approximately 40 weeks gestation, without complications. His neuromuscular development was initially normal, but at 4 years of age, there was a change in gait, with progressive loss of strength and frequent requests for being carried due to fatigue. By age 6, he lost the ability to walk, was unable to maintain an upright position, and experienced pain in the lower limbs, particularly in the joints, becoming wheelchair bound. He managed the wheelchair adequately, but was unable to perform transfers. Additionally, he developed mood changes, intense anxiety, nervousness, and easy crying. At 8 years of age (2020), he was referred to the medical genetics service. His main complaints included weakness in the lower limbs, joint pain in the knee and left elbow, recurrent upper respiratory infections (two to three episodes per month), abdominal pain and bloating, muscle weakness, difficulty moving limbs and trunk, inability to stand, intense pain in the thigh and calf, cramps, intense paresthesia, tinnitus in the right ear, asthenia, anxiety, irritability, and inappropriate crying.

In 2016, the patient underwent several tests: electromyography suggested myopathy but did not reveal peripheral polyneuropathy; muscle biopsy identified hypotrophic muscle bundles, suggestive of denervation; transthoracic echocardiogram was uneventful; spinal radiography showed a slight deviation of the thoracolumbar spine to the right, with no vertebral rotation and accentuated thoracic kyphosis and lumbar lordosis.

**Figure 1:**
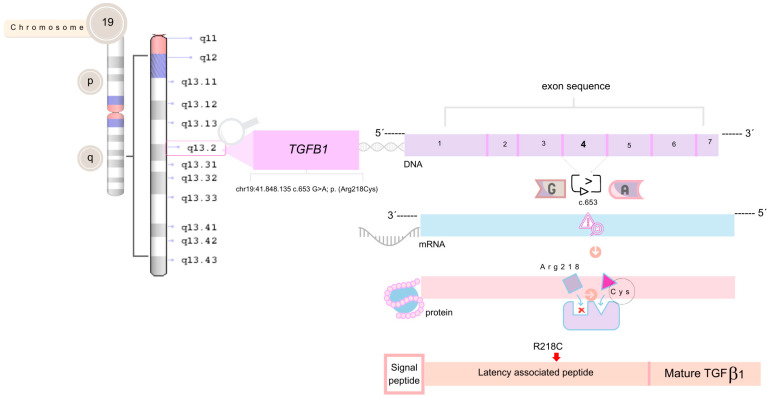
Representation of the missense variant in *TGFB1* at position chr19:41,848,135, consisting of the transition from guanine to adenine in exon 4, causing a substitution of arginine with cysteine at codon 218 located in the latency–associated peptide (LAP) domain.

In 2018, genetic testing for Spinal Muscular Atrophy (SMA) by Multiplex Ligation-dependent Probe Amplification (MLPA) reported no pathogenic variants of microdeletions or microduplications in exons 7 and 8 of the *SMN1* and *SMN2* genes. A Muscular Dystrophy Panel identified a missense variant (c.653G>A) in the *TGFB1* Gene (Figure 1), at chr19:41,848,135, resulting in p.(Arg218Cys) in heterozygosity. This variant was classified as pathogenic associated with CED according to the American College of Medical Genetics (ACMG) criteria.

In 2019, additional tests were conducted: audiometry and Transient Otoacoustic Emissions (TOAE) were normal. In 2022, an ophthalmological evaluation was performed with all tests within normal limits.

**Figure 2:**
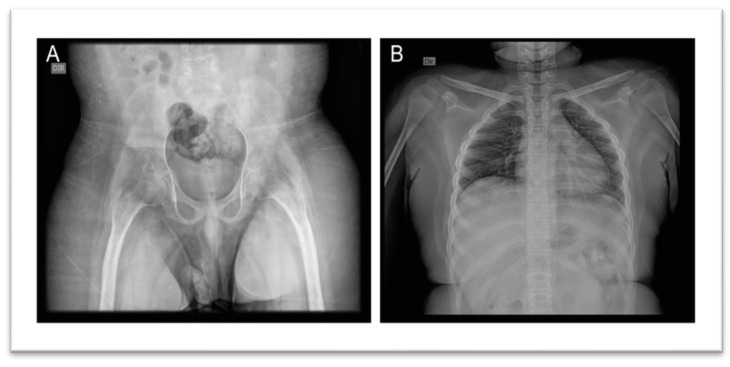
Radiographic Images. (**A**) Pelvis: Morpho structural changes in both hips characterized by verticalization and flattening of the acetabula, as well as cortical thickening of the femoral diaphysis bilaterally and apparent hypotrophy of the quadriceps muscles. (**B**) Spine: Slight deviation of the thoracolumbar spine to the right, with no vertebral rotation.

In 2024, blood and urine tests were conducted, including fasting glucose, urea, creatinine, uric acid, lipid profile, Aspartate Aminotransferase (AST), Alanine Aminotransferase (ALT), Creatine Kinase (CPK), potassium, sodium, glycated hemoglobin, Thyroid-Stimulating Hormone (TSH), Free Thyroxine (T4), Triiodothyronine (T3), vitamin B12, vitamin D, and folic acid, all within normal reference values. Hemogram results showed slightly low hemoglobin levels, 13.5 g/dL (reference: 14.0 to 18.0 g/dL) and lymphocytosis, 6400/µL (reference: 800 to 4000/µL). Urinalysis revealed dark yellow, slightly turbid urine, with a pH of 5.5, density of 1.028, traces of hemoglobin, hematuria (12,100/mL), presence of calcium oxalate crystals and mucus filaments. Thoracic radiographs were normal; pelvic radiographs showed morpho structural changes in both hips characterized by verticalization and flattening of the acetabula, as well as cortical thickening of the femoral diaphysis bilaterally and apparent hypotrophy of the quadriceps muscles (Figure 2A). Additionally, spinal radiographs described a slight deviation of the thoracolumbar spine to the right, with no vertebral rotation (Figure 2B).

In the most recent physical examination, conducted in 2024 at 11 years old, the proband exhibited the following phenotypes: weight 38 kg (p. 66%; +0.44 SD); head circumference 53.2 cm (p. 15%; −1.02 SD); broad and bulging forehead; oblique downward palpebral fissures; narrow nasal dorsum; medium nasal base; thin lips; short neck; symmetrical chest; symmetrical limbs with no deformities; lower limb areflexia; globally and symmetrically weakened lower limbs—muscle strength grade 2; active movement of upper limbs globally—muscle strength grade 4; presence of knee contractures, positive Thomas sign, significant thickening of tendons; and absence of spasticity.

The patient’s management involved multidisciplinary follow-up with specialists including a medical geneticist, neurologist, otolaryngologist, orthopedic surgeon, physiatrist, pulmonologist, ophthalmologist, occupational therapist, physical therapist, psychologist, and psychiatrist. Treatment with losartan was initiated but discontinued due to worsening the pain. Subsequently, corticosteroid therapy was prescribed, which improved pain and muscle strength, but was also discontinued because of significant weight gain, acanthosis, and fluid retention during the medication.

### 2.2. Patient No. 2 (Proband’s Mother)

In 2018, following the symptoms and positive test result of her second-born child, genetic testing of the mother, age 36, was conducted via a Muscular Dystrophy Panel, which revealed the same *TGFB1* variant found in her child. The mother reported the following symptoms: tinnitus in the left ear; recurrent upper respiratory infections with cough; difficulties with fine motor coordination; muscle weakness; fatigue; diffuse pain; myalgia; throbbing pain in thighs, legs, and arms; anxiety; tiredness; nocturnal awakenings due to pain; and dizziness. Despite her condition, she did not experience learning or developmental difficulties, maintained independent gait and daily living activities, and showed no tone alterations or limitations in joint range of motion. She is the third child of a non-consanguineous couple from the State of Goiás, Brazil. Prenatal care was performed, and she had a normal delivery with immediate postpartum, reporting only recurrent acute otitis media during childhood. No similar cases, genetic disorders, malformations, intellectual disabilities, mental illnesses, stillbirths, or neonatal deaths were reported in the family.

In addition to genetic testing, the patient underwent complementary examinations between 2023 and 2024, including cervical ultrasound, which identified submandibular lymph nodes with a normal appearance, without lymphadenopathy; audiometry, with hearing thresholds within normal limits in both ears except at 6 and 8 kHz in the left ear; and blood tests including hemogram, AST, ALT, urea, creatinine, sodium, potassium, calcium, iron, transferrin saturation index, phosphorus, TSH, and ferritin, all within normal reference values.

The phenotypic synopsis of the patient in 2024 at age 42 included weight 69.1 kg (p. 87%; +1.15 SD); head circumference 56.3 cm (p. 71%; +0.56 SD); broad forehead; oblique downward palpebral fissures; narrow nasal dorsum; medium nasal base; symmetrical chest; symmetrical limbs with no deformities; lower limb weakness; preserved extraocular motility; normotensive fundoscopic examination; photophobia; muscle strength in upper limbs grade 5; muscle strength in left lower limb grade 5; and proximal muscle strength in right lower limb grade 4 and distal muscle strength grade 5; fibroelastic lymph nodes in the submandibular and anterior cervical chains.

For adequate case management, the patient was referred to multiple medical and multidisciplinary specialties, including a physiatrist, medical geneticist, otolaryngologist, angiology specialist, ophthalmologist, neurologist, cardiologist, pulmonologist, nutritionist, physical therapy, speech therapy, and psychologist. Corticosteroid treatment was initiated but discontinued at the patient’s request due to weight gain, asthenia, and diarrhea reported after starting the medication.

### 2.3. Patient No. 3 (Proband’s Brother/Firstborn Child)

The proband’s brother, a 14-year-old male in 2024, was also diagnosed with Camurati–Engelmann disease (CED) following genetic testing for the same variant found in his younger brother. The diagnosis was made in 2018, along with that of his mother, at which time he was asymptomatic. However, the mother reported that he exhibited frequent falls and a toe-walking gait when he began walking at 14 months. Prenatal care was regular, and the pregnancy was uneventful with a term caesarean delivery. At 12 years old, in 2021, he reported the progression of the following symptoms: abnormal gait (bending the trunk and swaying); pain and cramps in the lower limbs (below the knees); weakness and difficulty walking and climbing stairs (requires support); burning sensation in the lower legs; and pulsatile temporal pain on the right. By age 14, in 2024, he also experienced pain and cramps in the upper limbs, particularly with exertion. His lower limb pain and cramps worsened with walking, and he exhibited symptoms of anxiety. Despite these challenges, the patient has appropriate speech and good academic performance.

Complementary tests performed in 2023 included Erythrocyte Sedimentation Rate (ESR), fasting glucose, sodium, potassium, phosphorus, chlorides, calcium, urea, creatinine, lipid profile, CPK, albumin, Anti-Streptolysin O (ASLO), rheumatoid factor, T3, free T4, TSH, ferritin, vitamin B12, folic acid, ionized calcium, urinalysis, and urine culture, all within normal reference values. However, blood tests revealed hemoglobin and hematocrit levels below reference values, 12.7 g/dL (reference: 14.0 to 18.0 g/dL) and 38.4% (reference: 40.0 to 50.0%), respectively; slightly elevated magnesium, 2.40 mg/dL (reference: 1.70 to 2.20 mg/dL); iron below reference values, 41 µg/dL (reference: 65 to 175 µg/dL); slightly elevated mucoprotein, 4.68 mg/dL (reference: 2.0 to 4.47 mg/dL); increased C-Reactive Protein (CRP), 12.4 mg/dL (reference: 0.0 to 5.0 mg/dL); and low vitamin D, 12.7 ng/mL (reference: >20.0 ng/mL).

**Figure 3:**
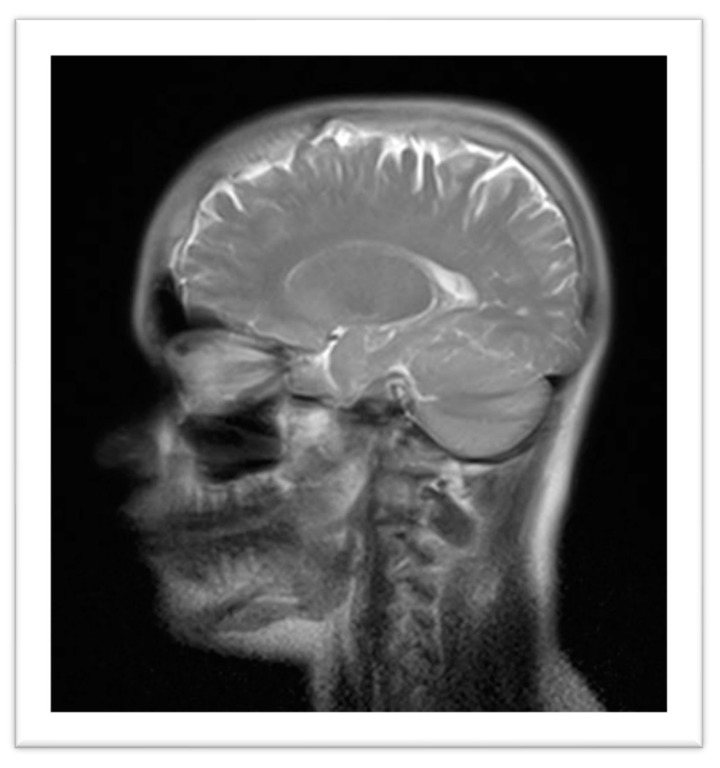
Brain Magnetic Resonance Imaging: calcification in the anterior aspect of the falx cerebri, hyperostosis of the inner calvaria table in the frontotemporoparietal regions, and asymmetry of the petrous apices.

**Figure 4:**
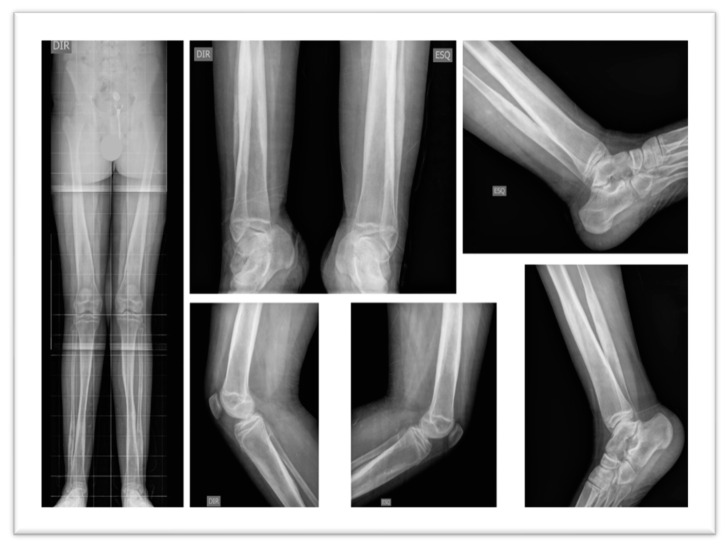
Radiographic Images of the pelvis, lower limbs, knees, and ankles: Bilateral cortical thickening of the diaphysis of the long bones with areas of apparent bilateral and symmetrical hyperostosis (mid/distal femur diaphysis, proximal and distal tibia and fibula).

Imaging studies included a Brain Magnetic Resonance Imaging (MRI), which revealed calcification in the anterior aspect of the falx cerebri, hyperostosis of the inner calvaria table in the frontotemporoparietal regions, and asymmetry of the petrous apices, with the right side showing an area of T2 hyperintensity, without expansive effect, contrast enhancement, or diffusion restriction, potentially indicative of a mucocele or effusion (Figure 3). Radiographs of the pelvis, lower limbs, knees, and ankles showed bilateral cortical thickening of the diaphysis of long bones with areas of apparent bilateral and symmetrical hyperostosis (mid/distal femur diaphysis, proximal and distal tibia and fibula), with no significant difference in limb length (Figure 4).

The phenotypic synopsis of the firstborn child, aged 14, included the following: weight 75 kg (p. 98%; +2.13 SD); head circumference 52 cm (p. 1%; −2.32 SD); reflexes present; genu varum; pes planus valgus; difficulty with orthostasis with support on toes and heels; able to transfer from lying to sitting without difficulty; slight difficulty in transferring from the floor to an upright position; difficulty walking on inclines and declines; iron-deficiency anemia; and vitamin D deficiency.

The patient was referred to various specialties including pediatric neurology, genetics, cardiology, pulmonology, otolaryngology, orthopedics, nutrition, physical therapy, psychology, and occupational therapy. Corticosteroid therapy was prescribed, but as of the time of this report, the results of the treatment are not yet available.

## 3. Discussion

This discussion centers on the genetic and clinical aspects of Camurati–Engelmann disease (CED) in a family with a missense variant (Chr19:41.848.135—c.653G>A—p.(Arg218Cys)) in the *TGFB1* Gene. This pathogenic variant involves a transition from guanine to adenine resulting in the substitution of arginine with cysteine at position 218. It is classified as pathogenic and codon 653 is one of the codons commonly involved in association with CED. As an autosomal dominant inheritance, carriers have a 50% chance of transmitting this variant to offspring [6].

ClinVar lists 18 pathogenic or likely pathogenic variants in *TGFB1*, with 11 being missense mutations [12]. According to Wallace and Wilcox [6], approximately 40% of CED cases are attributed to the p.(Arg218Cys) variant, while the second most common variant, p.(Arg218His), accounts for about 35% of cases.

*TGFB1* encodes a precursor complex consisting of a signaling peptide, a latency-associated peptide (LAP) region, and a mature TGFB1. Most disease-causing variants in CED, including p.(Arg218Cys), are activators of TGFB1 and cluster around the LAP region, crucial for protein dimerization. This results in a defective LAP molecule that poorly associates with the rest of the complex, leading to increased TGFB1 activity [13,14,15].

The arginine at position 218, located in exon 4 of the C-terminal LAP region, is a hotspot for mutations. According to Tao et al. [16], this amino acid is replaced in 60% of cases, with Wallace and Wilcox [6] reporting up to 75% of cases involving this substitution. McGowan et al. [13] found that the p.(Arg218Cys) variant increases *TGFB1* bioactivity, leading to elevated osteoclast formation in vitro. This heightened bioactivity causes imbalanced bone metabolism, a hallmark of CED.

Tao et al. [16] observed significant clinical variability even among individuals with the same disease-causing variant or within the same family. Those with the p.(Arg218Cys) variant showed more severe symptoms compared to those with the p.(Cys223Trp) variant, also located in exon 4 of the LAP region. This could be due to the frequency of the variant, as it is the most common cause of CED and may be inherited more frequently, potentially leading to genetic anticipation. Janssens et al. [17] suggested that there is a tendency for increased symptom severity in successive generations, supporting the hypothesis of anticipation.

Chen et al. [10] reported that all pathogenic variants increase *TGFB1* levels in circulation and bone, resulting in bone hyperplasia, accelerated bone turnover, inhibited normal bone mineralization, and decreased bone density. They noted that all mentioned variants, including p.(Arg218Cys), were located in highly conserved regions, highlighting their critical role.

The clinical phenotypes observed in the patients described here align with typical disease features. Imaging revealed cortical thickening of the diaphysis of long bones, present in 94% of CED patients. Other common symptoms included oscillating gait (48% of cases), extremity pain (68%), easy fatigability (44%), and muscular weakness (39%) [17].

Symptom onset varied among family members: the mother developed symptoms in adulthood, the firstborn during adolescence, and the second son in childhood. The severity of symptoms may relate to the age of onset or genetic anticipation, as evidenced by the more advanced symptoms in the child diagnosed early compared to the mother, who retained functional independence. Janssens et al. [17] observed that among 100 CED patients from 24 families, those with earlier onset tended to have more severe or both features, indicative of anticipation. The nature of the disease-causing variants did not favor anticipation.

The firstborn son presented with iron-deficiency anemia, with hemoglobin and iron levels below reference values. Li et al. [8] reported anemia in 39% of CED patients, aligning with this case. The patient’s low vitamin D levels are consistent with prior reports of vitamin D deficiency in CED patients [4,18,19]. Additionally, elevated C-Reactive Protein (CRP) was noted, consistent with findings from Restrepo and Molina [20].

CED treatment aims to alleviate symptoms, with corticosteroids, losartan, bisphosphonates, and NSAIDs being commonly used [11]. However, many patients experience limited improvement and adverse effects. For instance, losartan failed to relieve pain in the second child, and corticosteroid therapy resulted in significant weight gain, acanthosis, and fluid retention. Similar adverse effects have been documented, such as Cushing’s syndrome from corticosteroid treatment [19]. Moreira et al. [21] reported a case of a female patient with CED who developed ulcerative colitis. She underwent treatment with corticosteroids, which provided relief from pain. However, her treatment led to a dependence on higher doses. Subsequently, infliximab, a tumor necrosis factor-alpha (TNFα) inhibitor, was administered, which achieved remission of the ulcerative colitis and improved the pain associated with the disease.

Reports on CED are crucial for future management, diagnosis, and treatment of rare diseases. Advances in diagnostics and genetic counseling are needed for better patient outcomes.

## 4. Conclusions

The progression and onset of CED symptoms varied widely among patients, underscoring the need for precise clinical, genetic, and laboratory diagnostics for effective management and genetic counseling, especially given its rarity. It is not yet confirmed that the p.(Arg218Cys) variant causes a more severe phenotype, as symptoms ranged from mild to severe in the reported cases. The optimal treatment for CED remains unclear, and further research is needed to develop effective therapies that can improve patient quality of life and outcomes.

## Data Availability

Data are unavailable due to privacy or ethical restrictions.
